# Supplementary figures and images for: The effect of enhanced variability after performance stabilization through constant practice
Source: PeerJ. 2022 Sep 16;10:e13733. doi: 10.7717/peerj.13733 (PMC9484454; doi:10.7717/peerj.13733)

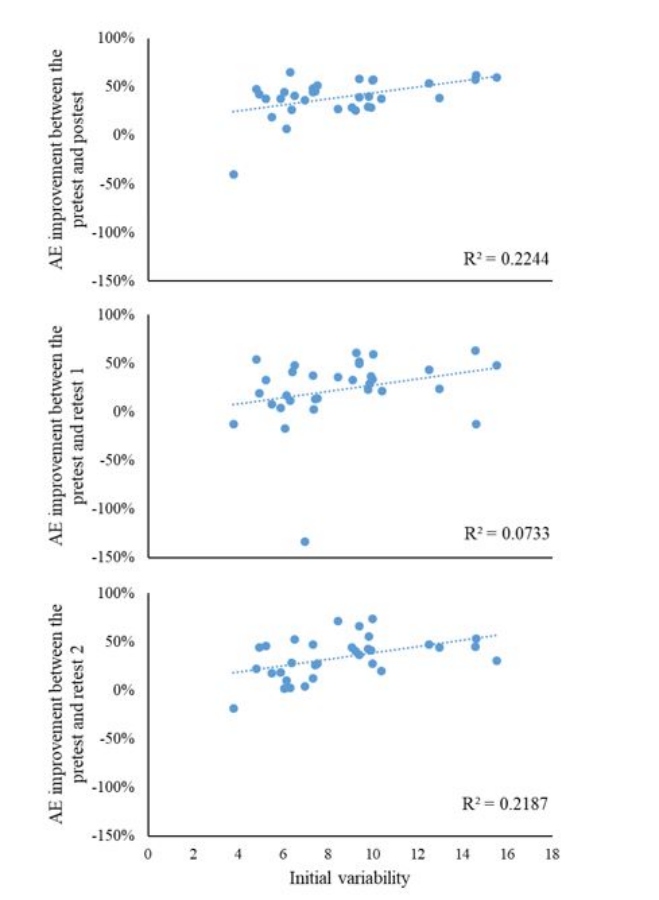

Supplement: Supplemental Information 1 [file peerj-10-13733-s001.jpg]
