# Supplementary material for: The effect of enhanced variability after performance stabilization through constant practice
Source: PeerJ. 2022 Sep 16;10:e13733. doi: 10.7717/peerj.13733 (PMC9484454; doi:10.7717/peerj.13733)
Supplement: Supplemental Information 2 [file peerj-10-13733-s002.docx]

| Supplementary table 1. Main effects of moment of testing in the variables measured in the two-way repeated measures ANOVA. | | | | | | | |
| --- | --- | --- | --- | --- | --- | --- | --- |
| **VARIABLE** | **Pretest** | **Post-test** | **Re-test1** | **Re-test2** | **F_3,87_** | **SIG.** | **η²** |
| AE | 7.80±2.61 | 4.46±1.22 | 5.68±2.54 | 4.86±1.61 | 26.426 | 0.000 | 0.477 |
| VE | 8.56±3.01 | 4.84±1.24 | 5.92±1.68 | 5.37±1.84 | 32.296 | 0.000 | 0.527 |
| Target to area | 8.47±6.09 | 5.47±1.58 | 5.67±2.33 | 4.77±1.32 | 7.573 | 0.005 | 0.207 |
| Ball to area | 8.77±6.27 | 5.20±1.42 | 5.38±1.68 | 4.78±2.15 | 8.701 | 0.003 | 0.231 |
| AE = Absolute error; VE = variable error. | | | | | | | |

| Supplementary table 2. Average values (mean ± SD) in each test of every variable calculated in the study for every training group and the **interaction (moment of testing*type of training practice)** results from the two-way repeated measures ANOVA. | | | | | | | | |
| --- | --- | --- | --- | --- | --- | --- | --- | --- |
| **VARIABLE** | **Training group** | **Pretest** | **Post-test** | **Re-test1** | **Re-test2** | **F_6,87_** | **SIG.** | **η²** |
| AE | Constant | 8.03±2.75 | 4.47±1.39 | 5.77±3.48 | 4.85±1.95 | 0.167 | 0.985 | 0.011 |
|  | Variable | 8.24±2.74 | 4.96±1.05 | 6.17±2.74 | 5.04±1.34 |  |  |  |
|  | Stabilized | 7.13±2.45 | 3.91±1.10 | 5.08±1.17 | 4.67±1.71 |  |  |  |
| VE | Constant | 8.36±2.37 | 5.07±1.30 | 5.42±1.96 | 5.35±1.91 | 0.595 | 0.685 | 0.039 |
|  | Variable | 9.22±3.57 | 5.14±1.18 | 6.38±1.80 | 5.30±1.48 |  |  |  |
|  | Stabilized | 8.01±2.95 | 4.31±1.21 | 5.83±1.30 | 5.46±2.28 |  |  |  |
| Target to area | Constant | 8.06±4.59 | 5.55±1.74 | 6.68±3.93 | 4.79±1.73 | 0.286 | 0.814 | 0.019 |
|  | Variable | 8.68±7.33 | 5.87±1.61 | 5.156±1.26 | 4.66±0.77 |  |  |  |
|  | Stabilized | 8.58±6.23 | 4.97±1.41 | 5.41±1.21 | 4.88±1.53 |  |  |  |
| Ball to area | Constant | 8.92±6.41 | 5.30±1.80 | 5.04±1.72 | 5.37±2.85 | 0.275 | 0.819 | 0.019 |
|  | Variable | 8.96±7.96 | 5.77±1.22 | 5.37±1.70 | 4.14±1.78 |  |  |  |
|  | Stabilized | 8.44±4.39 | 4.49±1.06 | 5.66±1.72 | 4.99±1.85 |  |  |  |
| AE = Absolute error; VE = variable error. | | | | | | | | |
