# Supplementary material for: The effect of enhanced variability after performance stabilization through constant practice
Source: PeerJ. 2022 Sep 16;10:e13733. doi: 10.7717/peerj.13733 (PMC9484454; doi:10.7717/peerj.13733)
Supplement: Supplemental Information 5 [file peerj-10-13733-s005.docx]

CODEBOOK FOR THE RAW DATABASE

Subject = Participant

Group:

- 1= Those subjects who followed constant training
- 2= Those subjects who followed variable training
- 3= Those subjects who followed constant training at first, and once they stabilized their performance, they started training variable.

Initial Variability :

- 1 = Low initial intrinsic variability
- 2 = High initial intrinsic variability

For the rest of the columns, all the variables are named with words formed by the nest parts:

1: MOMENT OF MEASURE

- Pre_ = Values corresponding to the pre-test series
- PreVar = Values corresponding to the constant training series prior to the variable ones (only in group 3 subjects)
- PostVar = Values corresponding to the first variable training series (only in group 3 subjects)
- Post_ = Values corresponding to the post-test series
- Retest1_ = Values corresponding to the first retention test series
- Retest2_ = Values corresponding to the second retention test series

2: OUTCOME

- M = Mean values of the measures
- SD = Standard Deviation values of the measures

3: VARIABLE MEASURED

- EAX = Absolute error (distance between the ball and the target).
- DM = Distance between the target and the delimited area in the moment of impact.
- BM = Distance between the ball and the delimited area in the moment of impact.

EXAMPLE:

PreVarSDEAX = SD value of the absolute error obtained by the participant in the constant training series before the application of variability (only in group 3 participants).
